# Achieving zero open defecation status through a community-based programme in Cavite, Philippines, 2013–2019: a field review

**DOI:** 10.5365/wpsar.2026.17.5.1323

**Published:** 2026-08-17

**Authors:** Julius R Migriño, Edroico Mari B Brillante, Maria Cecil Elisa G Esguerra, Orlie John Y Lavilla, Juan Miguel Lorenzo C Milla, Jennifer G Coritico

**Affiliations:** a San Beda University, Manila, Philippines.; b School of Medicine and Public Health, Ateneo de Manila University, Pasig City, Philippines.

## Abstract

**Problem:**

Open defecation is practised in several communities in the Philippines. Achieving zero open defecation (ZOD) certification in the Philippines has been shown to reduce disease burden and improve overall community health and well-being.

**Context:**

Philippine communities are guided by the Department of Health to achieve ZOD status through verified municipal inspections. The Barangay Anahaw II administrative unit was selected as a site for implementing sustainable, community-based health programmes aimed at achieving ZOD.

**Action:**

In 2013, a community diagnosis was conducted in the barangay to assess sanitation and health needs. In 2016, a multisectoral water, sanitation and hygiene committee, guided by a Community-Led Total Sanitation campaign, focused on improving the barangay’s sanitation status and promoting behavioural change. By 2017, the campaign’s activities had been implemented to prepare for and support ZOD verification and certification.

**Outcome:**

The community diagnosis in the barangay identified improper waste management, including limited access to toilets, as one of its top health concerns. The community-led campaign included strategies such as household inspections, assessments of the knowledge of heads of households, clean-up drives and incentives to encourage toilet construction. While an initial attempt at certification in May 2017 was unsuccessful, the barangay achieved provincial ZOD certification in July 2019.

**Discussion:**

A coordinated approach involving an initial community diagnosis, a multisectoral sanitation programme supported by local officials and the community, and continuing household engagement through activities and incentives enabled the achievement of ZOD certification.

## PROBLEM

Open defecation is the practice of defecating in fields, forests, bodies of water or other open spaces. (*1*) This practice is a public health issue because people are exposed to human excreta, which often contaminates drinking-water, exposing communities to pathogenic faecal microorganisms, and consequently to diseases. According to the World Health Organization/United Nations Children’s Fund (WHO/UNICEF) Joint Monitoring Programme for Water Supply, Sanitation and Hygiene, although open defecation rates decreased between 2000 and 2024, there are still several countries, including the Philippines, that have not yet eliminated open defecation. (*1*)

According to WHO, (*2*) four major health outcomes are attributable to poor water, sanitation and hygiene (WASH): diarrhoea, acute respiratory infections, undernutrition and soil-transmitted helminthiases. At least 1.4 million lives lost in 2019 could have been prevented with the use of safe WASH services. (*2*) A study in Kenya demonstrated a significant difference in the prevalence of diarrhoea between subcounties where open defecation was and was not practised. (*3*) According to the 2022 Philippine National Demographic and Health Survey, approximately 3% of households in the country, and up to 18% of households in rural areas, still practised open defecation. (*4*) Ending open defecation by 2030 is a key outcome target of Sustainable Development Goal 6 (clean water and sanitation). (*5*)

## CONTEXT

In the Philippines, local communities are classified into four sanitation levels: Grade 0 (open defecation), Grade 1 (zero open defecation), Grade 2 (basic sanitation) and Grade 3 (sustainable sanitation). Zero open defecation (ZOD), or Grade 1 status, is defined as a community wherein households have stopped practising open defecation and are using sanitary toilet facilities, and wherein no human faeces are openly visible or exposed to the environment. (*6*)

To reach ZOD status, seven criteria (*6*) must be achieved by the community: (1) open defecation is not practised; (2) 100% of households have access to sanitary toilets; (3) water and soap are available at or near toilets in all households; (4) excreta and diapers are disposed of properly; (5) a functional local government unit (LGU) coordinating body addresses WASH issues; (6) a local ordinance and a monitoring team help sustain ZOD status; and (7) an approved action plan or operational plan is in place and funding has been allocated to reach Grade 2 status. To be ZOD-certified, the community must pass several levels of verification, starting with a sanitary inspection by the rural health unit and moving up through municipal- and provincial-level certification. (*7*)

The province of Cavite is part of Region IV-A, in southern Luzon. Within Cavite, the municipality of Silang is a high-income, urbanizing municipality, classified as mainly agricultural and built-up areas. In 2020, its population was 295 644. The municipality is composed of 64 barangays (administrative units).

Barangay Anahaw II (BAII) in Silang (**Fig. 1**) consists of 768 households according to the 2015 census, with an average of 4.5 members per household. At least 80% of the families in BAII are classified as having income below the poverty threshold. Water is mostly provided by Silang Water District.

In June 2013, the San Beda University College of Medicine (SBU-COM) entered into a memorandum of agreement with Canossa Health and Social Center Foundation (CHSC), a Department of Health-recognized primary service institution situated in the barangay. The agreement aimed, in part, at developing sustainable community-based health programmes within BAII. The site was chosen from among 11 barangays in Silang due to its accessibility and readiness to take part in implementing such programmes. This study is a narrative analysis of the process of ZOD certification undertaken by BAII from 2013 to 2019.

## ACTION

### Community diagnosis

From June 2013 until March 2014, SBU-COM conducted a community diagnosis to assess the health needs of BAII. Health-related documents from the local rural health unit, containing health statistics about residents of the barangay, were reviewed, validated and correlated with the results of the community diagnosis. A total of 371 households were surveyed to determine their primary source of water, type of toilet facility, type of solid waste management and availability of basic sanitation facilities. Three barangay officials directly involved in health programmes were also interviewed by SBU-COM using structured interview guides to identify existing efforts to address sanitation issues in the barangay.

### Community-led total sanitation

Starting in May 2016, SBU-COM, CHSC and barangay officials established the BAII Multisectoral WASH Committee. The Committee guided a Community-Led Total Sanitation (CLTS) campaign that specifically aimed to (1) upgrade the level of sanitation of the barangay from Grade 0 to Grade 1; (2) improve residents’ sanitation and hygiene behaviours; and (3) educate residents about the health-related consequences of unsanitary practices.

An initial strategic plan was established by the Committee in October 2016, which included defining the roles and responsibilities of members, reviewing existing national and local sanitation laws, analysing the barangay sanitation situation and planning for specific campaign activities. Health education for heads of households was conducted by SBU-COM, CHSC and daycare teachers, while site visit validation of households without toilets was done by SBU-COM from October to December 2016. Additionally, a project called *Oplan Kubeta* (“Operation Toilet”) was launched in October 2016 to raise funds for the construction of household toilets. The Committee, through the barangay captain, also conducted regular reviews to assess the progress of the CLTS campaign.

### Zero open defecation verification

In preparation for the ZOD verification processes, SBU-COM and CHSC conducted spot inspections of households from October to December 2016 to assess the knowledge of block leaders (volunteer members of a barangay block) about the components of household ZOD certification; additionally, a participation-based cash incentive system for block leaders was established. Block leaders were also trained as hygiene educators by SBU-COM.

The initial verification assessment was conducted in May 2017. As part of the verification process, households were inspected using a checklist that assessed each household’s compliance with six sanitation requirements, ranging from the availability of soap and water to the proper disposal of different kinds of waste products (**Fig. 2**). Only households that fully met all six requirements passed the inspection. In December 2017, SBU-COM, CHSC and local officials launched the “4:30 PM habit,” a daily reminder delivered through a WASH song played over the public announcement system, with the aim of conditioning community members to continue practising water conservation, better sanitation and good hygiene.

In March 2019, to ensure sustained segregation, collection and disposal of waste, the barangay coordinated with the Municipal Environment and Natural Resources Office. Waste segregation seminars and participation in the national cleanest barangay awards were among the proposed strategies to sustain ZOD certification. The sustainability plans identified WASH Committee members as the responsible parties.

**Fig. 3** summarizes the community-based sanitation programme processes conducted by SBU-COM, CHSC and BAII from 2013–2019 to achieve ZOD certification.

## OUTCOME

The community diagnosis identified improper waste management as one of the key health-related concerns in the barangay. It revealed that 1% of households used pit latrines to dispose of human excreta, 6% did not have their own toilet, and 13% had a single toilet shared by about 10 people. Safe water was either insufficient or costly, worsening the barangay’s sanitation situation. Interviews with barangay officials in 2014 revealed that efforts in the community primarily focused on proper waste segregation and collection, proper disposal of animal waste and unclogging of canals; no community programmes had been established to address open defecation.

The WASH Committee identified three problems as the focus of the CLTS campaign: (1) the need for behavioural change; (2) improper waste disposal; and (3) unavailability of toilets in some households. The committee also proposed several activities in the implementation of the campaign, such as recognition of house certification through the awarding of stickers, strict implementation of existing sanitation laws, weekly clean-up drives, conduct of community sanitation self-assessment activities, and development of incentive mechanisms to address the unavailability of toilets, such as by providing consultations and construction materials. Two households were also chosen to be beneficiaries of the funds from *Oplan Kubeta*.

The first round of verification assessments occurred in May 2017, and the barangay failed the verification process due to improper diaper disposal in 10% of the households visited. To prepare for the second round of verification, seven rounds of house-to-house inspections were conducted by the WASH Committee’s hygiene educators from August to December 2017. During the inspection process, educators highlighted the six sanitation requirements evaluated by the checklist.

A second round of verification assessments occurred from November 2017 to April 2018. BAII passed the second verification and was qualified for ZOD certification from the rural health unit sanitary inspector. In May 2018, BAII was ZOD-certified by the Bulihan rural health unit and later certified at the municipal level. BAII was later provincially certified as having achieved ZOD status on 31 July 2019.

## Discussion

### Initial community diagnosis is a crucial first step

The community diagnosis implemented as the initial step of the process was crucial for its success. As a result, the barangay acknowledged that waste management was an important health concern, which directly led to the CLTS campaign. It also showed that at the LGU level, there were no established programmes to address open defecation. Community diagnosis, undertaken with LGUs and private partners, can help generate information that guides informed public health planning, enabling prioritization of resources for more effective programmes. (*8*)

### A multisectoral and collaborative programme was essential

The quick buy-in and direct involvement of local officials at all project stages also contributed to achieving ZOD certification. Active cooperation and participation of residents and ownership of the programme at the grassroots level also played crucial roles in the programme’s success. It is essential to involve local stakeholders at every step of the process to ground the programme in the context of the community, which is in line with the WHO WASH framework, (*1*) local studies (*9*) and guidelines. (*6*) A similar finding about the importance of a multisectoral approach came from a 2025 study in Zamboanga del Norte. (*10*)

Incentives for improvement of sanitation facilities were also helpful in achieving ZOD certification. The barangay providing construction materials for sanitary toilets and enabling households to consult with construction workers contributed to the achievement of ZOD. Total sanitation campaigns similar to CLTS have been found to improve sanitation facilities in communities, (*10*, *11*) while improved sanitation leads to better community health outcomes. (*2*)

### Continuous community engagement can facilitate certification

During the start of the project, a key challenge encountered was lack of participation by some households. A mid-project evaluation by SBU-COM showed that almost one third of the community was unaware of the CLTS campaign. This lack of awareness during the barangay’s initial verification attempt may have contributed to its failure. Kagayan recommends that to achieve ZOD outcomes, a programme’s aims and benefits to the community should be clearly articulated. (*9*) Different dissemination strategies may be effective in different contexts, such as policy briefs, social media and even consulting public relations experts. (*12*) Some of these strategies were implemented in this case, such as the 4:30 PM habit and household education sessions. Regardless of the strategies used, it is important to establish monitoring and evaluation systems to check an intervention’s progress in meeting its goals, in this case to encourage behavioural change.

Access to infrastructure and facilities is also important to translate behaviours into practice. (*13*) Despite being situated in a high-income municipality, BAII is a rural community with limited financial resources. Additionally, the barangay’s large population of low-income households and limited, costly and unreliable access to safe water were among other factors that may have hindered the initial ZOD initiative. (*9*, *14*, *15*)

### Limitations

This study has several limitations. Some reviewed reports were anecdotal and lacked quantitative measures, which limited the analysis. This study was also limited in scope, focusing only on documents used and processes implemented to achieve ZOD certification; BAII’s potential sanitation level progression from Grade 1 to Grade 2 was not reviewed due to shifts in the partnership’s priorities. Finally, BAII’s experience is specific to its context and may not be generalizable to other communities. It is recommended that similar programmes aimed at achieving ZOD status should develop both qualitative and quantitative metrics for monitoring and evaluation, (*8*) as well as assessing a community’s specific contextual barriers, such as poverty, water supply costs and demographics, all of which are important determinants of success. (*9*, *14*)

### Conclusions

The key lessons learned during BAII’s ZOD certification process included (1) the importance of an initial community diagnosis, which identified improper waste management and limited access to toilets as priority sanitation concerns; (2) the need for a collaborative sanitation programme that included the participation of private partners, local officials and residents; and (3) continuous household engagement throughout the verification process through activities such as knowledge assessments, clean-up drives and incentives. Similar communities can apply these general concepts, tailored to their own circumstances, resources and experiences to achieve ZOD status.
